# Thyroid Diseases and Thyroid Asymptomatic Dysfunction in People Living With HIV

**DOI:** 10.3390/idr14050071

**Published:** 2022-09-01

**Authors:** Cristina Micali, Ylenia Russotto, Benedetto Maurizio Celesia, Laura Santoro, Andrea Marino, Giovanni Francesco Pellicanò, Giuseppe Nunnari, Emmanuele Venanzi Rullo

**Affiliations:** 1 Unit of Infectious Diseases, Department of Clinical and Experimental Medicine, University of Messina, 98124 Messina, Italy; 2 Unit of Infectious Diseases, Department of Clinical and Experimental Medicine, University of Catania, 95131 Catania, Italy; 3 Biomedical and Biotechnological Sciences Department, University of Catania, 95131 Catania, Italy; 4 Department of Human Pathology of the Adult and the Developmental Age “G. Barresi”, University of Messina, 98124 Messina, Italy

**Keywords:** People Living With HIV (PLWH), thyroid dysfunction, thyroid diseases, HIV

## Abstract

Thyroid diseases (TDs) and thyroid asymptomatic dysfunctions (TADs) are correlated with Human Immunodeficiency virus (HIV) infection and Acquired ImmunoDeficiency Syndrome (AIDS) as well as many endocrine dysfunctions and dysregulation of hormonal axes. To date, available studies on People Living With HIV (PLWH) affected by thyroid diseases and asymptomatic dysfunctions are few and rather controversial. The purpose of the present non-systematic literature review is to recap the current knowledge on the main features of thyroid dysfunctions and disorders in PLWH. Large cohort studies are needed for a better comprehension of the impact, evolution and treatment of thyroid pathologies in the HIV-infected population.

## 1. Introduction

Thyroid hormones (THs) thyroxine (T4) and triiodothyronine (T3) are assembled in the thyroid gland and act peripherally, in a time and cell-specific manner, under the regulatory action of the deiodinases [1]. T3 and T4 regulate cell functions through a genomic (nuclear) and a nongenomic (non-nuclear) mechanism [2]. THs acts on almost all the nucleated cells and is essential for normal growth, brain development and energy metabolism [3,4]. Peripherally, the deiodinase 2 converts the pro-hormone T4 to T3, which is the biologically active form [1]. The thyroid-stimulating hormone (TSH) acts through its receptor (TSHR), stimulating both thyroid cell growth differentiation and function [5]. The circulating THs negatively feedback to the central components of the hypothalamic-pituitary-thyroid axis to maintain almost constant THs concentrations in blood circulation [6].

The TDs unbound to thyroxine-binding globulin (TBG) in serum are indicated as free T3 (fT3) and free T4 (fT4). Due to the complex inverse association between the pituitary-derived TSH and the circulating T4 and T3, TSH is the more sensitive marker of thyroid condition [3]. Thus, hypothyroidism is the conditions in which TSH concentrations in serum are above the range with fT4 levels below the normal range (in overt form), or with fT4 levels within the normal range (in subclinical form). Otherwise, hyperthyroidism is defined as TSH levels under the normal range with high fT4 levels (in overt form), or with normal fT4 (in subclinical form) [3].

Generally, hypothyroidism and hyperthyroidism develop from pathological processes that involve the thyroid gland (primary thyroid disease); however, rarely, they can rise up from dysfunctions of the hypothalamus or pituitary gland (central hypothyroidism) [3].

The autoimmune thyroid disorders (ATDs) include Graves’ disease (GD), Hashimoto thyroiditis (HT) and postpartum thyroiditis, and are characterized by the presence of circulating thyroid-specific auto-reactive antibodies that lead to axis dysfunction [3]. Thyroiditis, adverse effects of drugs, such as amiodarone and lithium, solitary or multiple autonomous nodules are also frequent causes of hyperthyroidism [3].

Despite the efficacy of the ART, HIV is still able to persist in a latent form and to escape the eradication [7]. HIV and AIDS are associated with many endocrine dysfunctions and dysregulation of hormonal axes [8,9,10,11]. TDs and TADs have been widely correlated with HIV infection, although the available studies about TDs and TADs in People Living With HIV (PLWH) are few and rather controversial [12]. To date, the available papers about TDs and TADs prevalence and distribution in PLWH suffer from the samples, definitions and outcomes heterogeneity. Furthermore, data about thyroid primitive and non tumors in seropositive patients are almost completely lacking [12].

The aim of this review is to evaluate the current knowledge on the epidemiology, pathogenesis, clinical and laboratory features, diagnosis and treatment of TDs and TADs in PLWH.

### 1.1. Epidemiology

The prevalence of thyroid dysfunctions in PLWH is similar to that in the general population. However, subclinical TDs, defined by an abnormal serum levels of thyrotropin or thyroid stimulating hormone (TSH) with normal peripheral THs concentrations, such as sick euthyroid syndrome (SES), subclinical hypothyroidism or hyperthyroidism and isolated low T4 levels, are more frequent in PLWH [8,13,14].

It is estimated that approximately one-third of PLWH have biochemical alterations of thyroid function, and in about 1% to 3% of cases they develop overt TDs [15].

Different studies have analyzed the distribution of thyroid clinical and subclinical dysfunctions, and it was found that the most prevalent TD in PLWH seems to be hypothyroidism, prevalently subclinical. In a multicenter study in northern France, on 350 HIV patients, 16% of them was found to have hypothyroidism, 6.6% subclinical and 2.6% overt, and 6.8% was found to have isolated low fT4 level [16]. Similarly, in another recent cross-sectional study in Chile on 127 HIV patients, the authors revealed a prevalence in hypothyroidism (10.2%), followed by hypothyroxinemia (6.29%) and reported only one case of hyperthyroidism. Notably, the thyroid abnormalities biochemically detected were generally asymptomatic [17]. Conversely, an Italian retrospective study on 6343 PLWH found 113 patients (1.8% of the total) who have received a diagnosis of symptomatic thyroid diseases (10 patients with euthyroid goiter were excluded): 81 of them (71.7%) with hypothyroidism, 21 (18.5%) with hyperthyroidism and 11 (9.7%) with a primary thyroid tumor. Particularly, 63 out of 81 patients with symptomatic hypothyroidism (77.8%) suffered from HT, while 17 out of 21 patients with symptomatic hyperthyroidism (80.9%) were affected by GD [12].

Apart from being the most prevalent thyroid condition, subclinical hypothyroidism is less frequent in male seropositive patients than in female ones [12,18]. Moreover, the prevalence of this condition in male PLWH is higher than in male general population [18].

Concerning antiretroviral treatment, few data are available. An Italian retrospective analysis on 687 seropositive patients who were receiving (90.2%) and not receiving (9.8%) ART, observed that subclinical hypothyroidism had a similar prevalence in both groups. Moreover, the authors did not find any statistically significant relationship between any drugs and subclinical hypothyroidism [18].

Concerning the geographical distribution of TDs, many of them are connected to the deficiency in many regions of iodine, an integral element in the structure of THs. Nodular thyroid disorders are more frequent in areas of the world where there is iodine shortage. On the contrary, autoimmune TDs, such as HT and GD, usually occur in iodine-replete populations [3]. About one-third of the world’s population lives in areas of iodine deficiency, and a particularly risky population are people living in the mountainous areas in South-East Asia, Latin America and Central Africa [19]. Thus, the correct assessment of nutritional status and the supplementation of deficiencies of the micronutrients is even more fundamental in PLWH coming from an iodine deficiency country [20].

### 1.2. Pathogenesis

The opportunistic infections act as triggers of immune activation and this seems to be at the basis of the pathogenetic mechanisms of TDs in PLWH, where the HIV infection is accompanied by the development of inflammatory and neoplastic processes in the thyroid gland [21]. A characteristic of HIV infection in thyroid gland is an early lowering of reverse triiodothyronine (rT3) levels, with normal fT3 levels that successively develop in an isolated low fT4 level [15].

Several studies have shown the inverse correlation between the lymphocytes T CD4+ (CD4) counts and serum TSH levels, emphasizing the trend for hypothyroidism as HIV disease progression in both adults and pediatric cases [22,23]. Moreover, there is substantially lower CD4 count in PLWH with subnormal levels of fT4 compared with those with normal fT4 levels [24]. Thus, the authors hypothesize to use fT3/fT4/TSH serum levels as surrogate markers of the progression of HIV disease [22,23].

IL-2 is the main cytokine that induces T cell activation and differentiation. Thus, in 2003, it was hypothesized a correlation between CD4+ counts and fT4 partly mediated by IL-2. The infusion of IL-2 in asymptomatic PLWH resulted in transient increases in TSH, fT4 and CD4l counts, supporting an interaction between the pituitary–thyroid hormones and the immune system, today not fully clarified [24].

Otherwise, TDs in PLWH might be the result of a direct cytopathic effect of HIV on the thyroid gland, in addition to the adverse effects of antiretroviral therapy (ART) [25]. As observed on the renal function [26], several studies have documented the association between the use of ART and the increase of thyroid dysfunction in PLWH. For example, the use of nucleoside reverse transcriptase inhibitors (NRTI), such as stavudine, and of non-nucleoside reverse transcriptase inhibitors (NNRTI), such as efavirenz, seems to be associated to an increased risk of hypothyroidism [13,27,28,29]. The mechanism is still uncertain, but perhaps the antiretroviral drugs interfere directly with the synthesis and/or the catabolism of THs, or otherwise they could hamper hormone releasing from the thyroid gland [30].

Concerning the role of the ART on the thyroid abnormalities, there is much evidence about the dysregulation of the autoimmune response as a consequence of the immune reconstitution syndrome (IRIS) after the beginning of the ART, leading to ATDs [13,31]. About 1–2% of PLWH develop GD [15].

### 1.3. Screening

To date, there is insufficient evidence to routinary recommend the assessment of thyroid function in asymptomatic PLWH [13,31]. However, some groups seem to be particularly risky populations for TDs, such as young seropositive overweight women affected by diabetes mellitus, and PLWH with poor lipemic control, in which thyroid screening could be indicated even without symptoms [32]. Moreover, considering the higher incidence of thyroid neoplasia in people with family history of thyroid tumors, some authors agree on the regular examination of this risky group in order to ensure early diagnosis and treatment, eventually implementing the ultrasound screening [33,34].

## 2. Subclinical and Overt Hypothyroidism

Subclinical hypothyroidism—or compensated hypothyroidism or mild hypothyroidism—is a thyroid abnormality characterized by elevated TSH serum levels and normal FT4 serum levels [35].

Subclinical hypothyroidism is the most common thyroid abnormality in both the general population and PLWH, with a large prevalence seronegative elderly women [14,25] and in the pediatric seropositive population [36]. It is more common in PLWH on ART and with lower CD4 count [32,37]. The main predictors of subclinical hypothyroidism in PLWH with severe immunodeficiency at HIV infection onset, seem to be thyroid peroxidase (TPOAb) detection and tuberculosis co-infection [38]. Poor lipemic control, and specifically hypothyroidism, also induces hypercholesterolemia, which is a risk factor for subclinical hypothyroidism [32,39].

The pathogenic features of subclinical hypothyroidism are still not completely understood; however, TBG seems to be implicated in some forms as well as higher TSH levels are associated with a more progressive disease [36]. It seems that in the case of severe immunocompromisation, the thyroid response and functions are impaired by an increase in the concentration of TBG [36].

In up to 40% of cases, the thyroid axis normalizes spontaneously; nonetheless, in about 4% of cases per year the subclinical hypothyroidism proceeds to clinical hypothyroidism, especially in presence of TPOAb (in about 2% per year in absence of TPOAb) [35].

Overt hypothyroidism is quite rare in PLWH and even more so in children living with HIV (CLWH) [36]. The manifestations are the same than in the general population, characterized by an insidious and progressive symptomatological cortege of fatigue, weakness, dry skin, cold intolerance, mental slowdown, constipation, hoarse voice, paresthesia, bradycardia and delayed relaxation of tendon reflexes [40]. The main differences between subclinical and overt hypothyroidism are summarized in Table 1.

Weak evidence supports the administration of levothyroxine (LT4) in subclinical hypothyroidism to ameliorate the symptoms [35]. According to many guidelines, the indication for treatment is with detection of TSH > 10 mIU/L. Moreover, the European Thyroid Association (ETA) guidelines suggest to consider treatment even in the case of repeated finding of TSH between 5 and 10 mIU/L, and in the case of symptoms compatible with hypothyroidism [41].

In people with cardiovascular risk and laboratory diagnosis of subclinical hypothyroidism, an early beginning of LT4 treatment might apport a benefit; however, in other individuals without clinical manifestations and TSH < 10 mIU/L, a wait-and-see strategy for spontaneous normalization of thyroid function is advocated. Furthermore, in mild hypothyroidism, if there is normalization of TSH serum levels without clinical improvement, it is advocated to stop the treatment [41].

## 3. Subclinical and Overt Hyperthyroidism

Subclinical hyperthyroidism is a condition characterized by a low TSH serum level concentration, with normal fT4 and T3 or fT3 serum levels, in the absence of hypothalamic or pituitary disease, or ingestion of drugs that interfere with TSH secretion such as glucocorticoids or dopamine [19,42]. Subclinical hyperthyroidism in the general population is estimated to be about 1% to 2%, more present in the iodine-deficient areas because of the functional autonomy from nodular goiters [19,42]. The risk of progression to overt hyperthyroidism is higher in people with TSH < 0.1 mIU/L than in people with low but detectable TSH levels [40,42].

While overt hyperthyroidism manifests itself with irritability, heat intolerance, sweating, warm moist skin, palpitations, tachycardia, fatigue, weight loss with increased appetite, diarrhea, tremor, muscle weakness, hyperreflexia and lid retraction, subclinical hyperthyroidism is characterized by a reduced bone mineral density and an increased risk of atrial fibrillation as high as the degree of thyroid hyperfunction [43,44]. The main features of subclinical and overt hyperthyroidism are reported in Table 2.

Generally, subclinical hyperthyroidism is spontaneously resolved; thus, it is advocated to repeat serum TSH, T3 and T4 serum levels after 3–6 months, before confirming a diagnosis and eventually starting a treatment [44]. The American Thyroid Association and the American Association of Clinical Endocrinologists suggest the treatment when TSH serum levels are <0.1 mIU/L, in patients > 65 years old or with comorbidities (heart pathologies or osteoporosis), but the effectiveness of treatment is still unclear [42].

## 4. Sick Euthyroid Syndrome

Sick euthyroid syndrome (SES), also known as nonthyroidal illness syndrome (NTIS), is a condition characterized by a consistent decrease in serum fT3 levels with normal fT4 and TSH levels and increased concentration of serum rT3 [45,46]. SES is frequently observed in euthyroid patients with severe critical illness, who underwent deprivation of calories, following major surgeries and in an advanced stage of HIV disease [30,46]. Several studies have shown that NTIS has a clear correlation with morbidity and mortality in severely ill patients [47,48].

A recent retrospective study in Somalia enrolled 976 patients admitted to the internal department with thyroid disorders. Among them, 90 patients (9.2%) were seropositive (47 have HIV and 43 have HIV + malaria). Out of 90 seropositive patients, 57 (63.3%) have SES, while among seronegative patients the prevalence of SES was 58% (514/886) [49].

Although the pathogenetic mechanism of SES is not completely understood, it is probably related to a hypothalamic–pituitary deficit due to the progression of immunodeficiency and the progress of cachexia [30]. Many factors are involved in the development of SES, including alterations in the activity of type 1 and 3 deiodinases, thyrotropin-releasing hormone (TRH) and TSH secretion, hormone binding to plasma proteins, thyroid hormone transporter expression and activity, and the thyroid hormone nuclear receptor complex [50].

From a recent analysis of the incidence of SES in Intensive Care Unit (ICU) in China, some elements were identified as independent risk factors for NTIS, such as brain natriuretic peptide (BNP), platelets (PLT) and albumin [51].

SES appears to be a physiologic adaptation in response to a pathological status; thus, the treatment to restore normal serum levels of THs in order to impact positively on prognosis and outcome, to date, is the focus of many studies; however, there is not yet a clear evidence of benefit [52]. In some people with SES, interesting data suggest a possible role for the infusion of hypothalamic-releasing factors which can reactivate the thyroid axis, inducing an anabolic response [50,53]. However, to date no consensus exists on therapeutic intervention for SES [54].

## 5. Autoimmune Thyroid Diseases

After many years from ART introduction, the incidence of autoimmune diseases in PLWH is progressively increased [55].

In the development of autoimmune diseases in PLWH, several interleukin (IL) seem involved, such as IL-6 which serum levels are found higher in people with autoimmune disorders and might be used as early predictor of this kind of diseases [56]. Concerning ATDs, apart from larger amounts of IL-6, increased levels of pathogenic Th17 and Th22 cells seem involved in the pathogenesis of ATDs, as suggested by the detection of Th17 and Th22 cells in the thyroid glands in people with HT [57]. Many microorganisms are considered involved in the pathogenesis of ATDs, including Yersinia enterocolitica, Helicobacter pylori, Borrelia burgdorferi, Coxsackie virus HTLV-1 and HIV [58].

However, most of the reported cases of ATDs in PLWH regard the development of a pathological autoimmunity against thyroid gland following the ART. The immune reconstitution therapy (IRT) leads to about 88% of GD, 6% of HT and 6% of hypothyroidism [59].

### 5.1. Graves’ Disease

GD is an autoimmune disorder sustained by the abnormal production of autoantibodies to the TSHR, called TSHR autoantibodies (TRAb). TRAbs act as agonists of TSHR, inducing THs oversecretion, releasing the thyroid gland from pituitary control [60]. Biochemically, GD is characterized by low TSH and elevated fT4 levels [31].

GD is the most reported manifestation among ATDs associated with IRIS [61]. IRIS is a condition characterized by a rapid recovery of CD4 following the initial depletion phase, after the beginning of ART. It is well described in the setting of opportunistic infections and tumors in PLWH, such as tuberculosis and Kaposi sarcoma [62,63]. However, an IRIS can occur due to an immune response against self-antigens, leading to autoimmune diseases such as rheumatic diseases, multiple sclerosis and GD.

GD associated with IRIS (IRIS-GD) is more common in women (3.0%) than in men (0.2%), with a high frequency among black Africans populations [59]. The onset of GD-IRIS occurs between 8 and 33 months after the beginning of ART, with high variability [59]. In an anecdotal case, the authors observed a repeated presentation of GD, the second episode after a long period, when complications due to ART were not expected and the patient was apparently in good conditions [64].

Although the pathogenic mechanism of GD-IRIS is not completely known, it seems to be a consequence of an immunoregulatory disequilibrium, characterized by thymic enlargement, failure of thymic deletion of autoreactive T-cells, persistence of T-cell receptor excision circle levels in CD4 and lymphocytes T CD8+ (CD8), and high circulating levels of naïve CD8 [59,65]. It seems that immune reconstitution promotes a profile shift in cytokines production from TH2 to TH1 types, permissive for the development of autoimmunity events [66].

It is unknown the exact incidence or prevalence of Graves’ orbitopathy known as thyroid eye disease (TED), which is rarely seen as a manifestation of an IRIS in PLWH [67]. The pathogenesis of TED involves complex and not fully understood interactions between genetic and environmental factors. Those interactions begin and propagate an inflammatory cascade, involving insulin-like growth factor 1 receptor (IGF1-R) and TSHR autoantibodies causing the characteristic retro-orbital tissue expansion and inflammation [60,67].

Substantially, the treatment options of GD were not changed during the time, and are represented by antithyroid drugs, radioiodine and/or surgery [60]. In the general population, the first-line treatment of GD is medical with antithyroid drugs such as thionamide, carbimazole, methimazole, or propylthiouracil in rare cases effective in controlling the hyperthyroidism [68,69]. Radioactive iodine (RAI) treatment is an increasing therapy used either as first-line treatment or as an alternative treatment in patients who do not tolerate or respond to medical therapy. RAI treatment is based on the principle that the thyroid gland entraps iodine and uses it for THs production; thus, once inside the thyroid follicular cells, RAI releases energetic beta particles, leading to the destruction of the follicular cells [65]. A recent retrospective study showed that there is no difference in response to RAI treatment between PLWH with IRIS-GD and GD in HIV-uninfected patients [65]. Surgical approach is a valid option; however, it can determine hypoparathyroidism or laryngeal nerve damage [60].

### 5.2. Hashimoto’s Thyroiditis

HT is the most common cause of hypothyroidism in the United States [70]. Although the pathogenetic mechanisms are not completely clarified, complex interaction among genetic elements, environmental factors and epigenetic influences are involved [71].

Clinical manifestations of HT coincide with primary hypothyroidism and are determined by the progressive damage to the thyroid gland mediated by the abnormal production of antibodies against thyroid gland, TPOAb and thyroglobulin (TGAb) [71]. Sometimes, HT in PLWH may present as an acute and painful thyroiditis with an initial phase of thyrotoxicosis, accompanied by elevated titer of TPOAb, followed by a phase of persistent hypothyroidism [72]. Rarely, an undiagnosed HT leads to an accumulation of effusive fluid into the intrapericardial space and to a cardiac tamponade [73].

Besides this, several studies in the general population have suggested that a relationship between HT and malignant transformation, through immunological or hormonal pathways, is still unknown, thus needing further investigations [71].

The diagnosis of HT is based on the presence of autoantibodies, such as TGAb and TPOAb, that have been used to diagnose it. In the general population, about 10% of people with positive TGAb and/or TPOAb have hypothyroidism. On the contrary, the presence of a blocking antibody known as TSH-stimulation blocking antibody (TSBAb) leads to thyroid atrophy and hypothyroidism [74].

The treatment of the HT is a long-life substitution therapy for hypothyroidism, although cases of spontaneous recovery have been reported [74].

### 5.3. Hypothyroidism after IRT

The hypothyroidism developing after IRT is generally potentially reversible, in contrast to the hypothyroidism of HT, which is irreversible. After IRT, in settings such as multiple sclerosis following alemtuzumab treatment, or allogeneic bone marrow/hematopoietic stem cell transplantation, TRAb are positive in about 70% of cases, indicating the potential later recovery from hypothyroidism, because TBAb usually do not persist for a long time. On the contrary, in PLWH, hypothyroidism is not associated with detection of TPOAb/TRAb positivity, suggesting a non-autoimmune pathophysiology, that is, infectious thyroiditis [59].

## 6. Infectious Thyroiditis

Before the advent of ART, PLWH were at high risk of infectious TDs such as acute or subacute thyroiditis or, rarely, suppurative thyroiditis. Generally, thyroiditis develops either from adjacent sites of infection or after hematogenous spread from a distant site as part of a disseminated infection, in the contest of septicemia by opportunistic pathogens such as Mycobacterium tuberculosis, Mycobacterium avium, Cytomegalovirus, Cryptococcus neoformans or Pneumocystis jiroveci [15,59].

Due to the gland’s generous blood supply and lymphatic drainage, and the antimicrobial action of iodine, suppurative thyroiditis is quite uncommon among thyroiditis [75]. Generally, it is associated with congenital malformation as pyriform sinus fistula or underlying thyroid disease, especially multinodular goiter, and HIV infection is one of the most common predisposing factors for its development [75,76]. Streptococcus, Staphylococcus, Pneumocystis jirovecii and Mycobacterium, are the most common agents responsible for suppurative process in thyroid gland [76], in general characterized by dysphagia, fever and the presence of a tender thyroid mass [75]. In case of opportunistic infections clinical presentation may be more insidious [75].

Effectively, seldom does infectious thyroiditis present itself with clinical manifestations; however, some cases of thyroiditis with thyrotoxicosis or hypothyroidism due to Pneumocystis jirovecii have been reported [77]. In the case of overt clinical presentation, thyroid ultrasound and fine-needle aspiration cytology are indicated [15,59]. The management of thyroiditis includes the administration of appropriate antibiotics and, especially for the suppurative forms, the drainage of any abscesses [75,76].

## 7. Thyroid Neoplasia

In the post-ART era, there was a decrease in the incidence of AIDS-defining cancers (ADCs) and a contemporary increase in others such as melanoma, anal, cervix, liver, breast and prostate cancers [78,79,80,81,82,83,84,85]. However, PLWH are frequently diagnosed with advanced-stage Non AIDS-defining cancers (NADCs) and experience a more aggressive clinical course, poorer outcome, higher rate of relapse and worse treatment response than the general population [33]. Screening tests applied for an early detection of tumors in PLWH are the same used in the general population; however, the real challenge is to improve access and adherence to screening tests for cancers in this population [86,87]. It is commonly observed a suboptimal adherence to screening test by PLWH, and, often, seropositive patients come to a doctor’s attention only in the presence of symptoms (e.g., loss of weight, asthenia). Screening programs for neoplasia are crucial to implement the chance of survival, because PLWH are often diagnosed at an advanced stage and are affected by more aggressive forms of tumors than the general population (e.g., HPV-related head and neck squamous cell carcinoma (HNSCC), bladder tumor) [86,87].

Nevertheless, concerning thyroid neoplasia, to date there is no evidence of an increased risk of malignancies either in comparison to the general population or after IRT. Thus, considering the huge prevalence of thyroid nodules in the population and the very low risk of thyroid cancer, the European Thyroid Association Guidelines recommend against routinary perform of thyroid US in PLWH on ART because of concrete the risk of overdiagnosis and overtreatment [59].

Globally, in the past three decades, there was a substantial increase in the incidence of thyroid tumors of all sizes and stages, apparently due to an increased surveillance and also an increasing incidence-based mortality for papillary thyroid cancer [88,89]. In the general population, in China, the age-standardized incidence of thyroid neoplasia is 9.61/1,000,000 [90]. In a retrospective study conducted on PLWH admitted to the hospital with cancer, in China from 2007 to 2020, thyroid neoplasia was the second most common NADCs. Precisely, 17 out of 200 patients (8.5%) were diagnosed with thyroid neoplasia while 38 (19%) had lung cancer [33]. Papillary thyroid cancer is the most common type of thyroid tumor (82%) in the adult and the pediatric population both in PLWH and in the general population, followed by follicular (7%) carcinoma, medullary thyroid tumor (3%) and anaplastic tumor (1%) [49,88,89,91]. In the study of Properzi et al., out of 11 PLWH with thyroid cancer, medullary thyroid cancer occurs in 2 patients (18.18%) [12].

Many studies suggest that the female sex, the thyroid neoplasia family history and the elevated BMI are associated with a higher risk of neoplastic transformation in the thyroid gland [34,92,93] Recently, it was also proposed a role for IL-32, which seems involved in different cancers, including thyroid neoplasia and which seems downregulated in several inflammatory conditions from neuronal diseases and metabolic disorders to HIV infection [94].

Usually, the thyroid cancer presents as a solitary nodule or manifests in increasing goiter size [19].

Treatments for thyroid cancers are the same as in the general population; however, close cooperation in a team with oncologists and surgeons is mandatory for the correct management of the patient, taking into account any eventual drug–drug interaction between chemotherapeutic and antiretroviral agents that could either increase drugs toxicity or decrease their efficacy [33].

## 8. Conclusions

TDs, TADs and thyroid tumors are not very common in PLWH. To better characterize the pathogenetic mechanism and the precise role of HIV infections in their development, further studies are needed. Although there is no necessity of screening PLWH without suggestive symptoms, it might be useful for some risky groups, such as women with family history of thyroid tumors and people affected by diabetes mellitus or poor lipemic control.

Moreover, the role of ART in the development of thyroid abnormalities is still not fully comprehended and needs more studies. There is little evidence about the dysregulation of the autoimmune response as a consequence of the IRIS and, in particular, on the use of stavudine or efavirenz. However, other studies did not observe significant differences between PLWH on ART and ART-naive. It is our opinion that this incongruity is probably due to a lack of knowledge about the precise role of the ART in the pathogenic mechanism of thyroid pathologies. To conclude, in the presence of TDs, TADs or thyroid tumors, a close collaboration with endocrinologists and, eventually oncologists, is desirable for a complete management of these patients.

## Figures and Tables

**Table 1:** Main differences between subclinical and overt hypothyroidism.

	Subclinical Hypothyroidism	Overt Hypothyroidism
Prevalence on PLWH	The most common TDs	Quite rare
THs	↑TSH and normal FT4	↑TSH and ↓FT4
Clinical manifestations	Quite rare, are the same than in the overt hypothyroidism	Fatigue Weakness dry skin cold intolerance mental slowdown constipation hoarse voice paresthesia bradycardia delayed relaxation of tendon reflexes
Treatment	Yes, if TSH > 10 mIU/L and/or in case of symptoms compatible with hypothyroidism and/or in presence of TPO antibodies	Yes

Differences between subclinical and overt hypothyroidism. ↑ elevated; ↓ low [36,37,38,39,40].

**Table 2:** Main differences between subclinical and overt hyperthyroidism.

	Subclinical Hyperthyroidism	Overt Hyperthyroidism
THs	↓TSH and normal FT4	↓TSH and ↑FT4
Clinical manifestations	reduced bone mineral density increased risk of atrial fibrillation	Irritability heat intolerance sweating warm moist skin palpitations and tachycardia fatigue weight loss increased appetite diarrhea tremor muscle weakness hyperreflexia lid retraction
Treatment	Yes, when TSH < 0.1 mIU/L, in patients > 65 years old or with comorbidities (heart pathologies/osteoporosis)	Yes

Differences between subclinical and overt hyperthyroidism. ↑ elevated; ↓ low [41,42,43].
